# Microplastics in Drinking Water: A Review of Sources, Removal, Detection, Occurrence, and Potential Risks

**DOI:** 10.3390/toxics13090782

**Published:** 2025-09-15

**Authors:** Ting Cai, Zhihe Tang, Tao Gu, Kun Tong, Xinwei Wang, Hao Chen, Xingnan Zhou, Zi Long, Chunmei Hao, Chunmao Chen, Rong Zeng

**Affiliations:** 1 CNPC Research Institute of Safety and Environmental Technology, Beijing 102206, China; 2 College of Chemical Engineering and Environment, China University of Petroleum-Beijing, Beijing 102249, Chinac.chen@cup.edu.cn (C.C.); 3 MOE Key Laboratory of Pollution Processes and Environmental Criteria, College of Environmental Science and Engineering, Nankai University, Tianjin 300350, China; chenhao@nankai.edu.cn; 4 CNPC Changqing Oilfield, Xi’an 710018, China

**Keywords:** MPs, drinking water, detection techniques, removal strategies, potential risks

## Abstract

The emergence of microplastics (MPs) in drinking water supply systems has become a significant environmental challenge. Although the potential impacts of MPs in drinking water on human health remain incompletely understood, the ingestion of MPs through drinking water has raised substantial public concern regarding health risks. This review synthesizes contemporary scientific advances focusing on the following: I. the sources and fate of MPs in drinking water supply chains; II. comparative assessment of removal at treatment; III. detection techniques based on microscopy, spectroscopic, and thermal methods; and IV. the potential hazards of MPs to human health. This study aims to provide novel insights into understanding the threats posed by MPs in drinking water and to facilitate the development of effective monitoring strategies.

## 1. Introduction

Plastic products are extensively utilized due to their advantageous properties, including their superior waterproofing and moisture resistance, cost-effectiveness, chemical stability, and electrical insulation. Global plastic production reached 3.907 × 10^8^ tons in 2021, of which 90.2% comprised fossil-based plastics, 8.3% were post-consumer recycled plastics, and 1.5% were bio-based/biodegradable plastics [1]. In addition to escalating production and consumption, the mismanagement of plastic waste exacerbates environmental plastic pollution. Global plastic production is projected to reach 3.3 × 10^10^ tons annually by 2050 [2]. In aquatic systems, plastics undergo degradation into microplastic particles. Microplastics (MPs) are defined as solid plastic particles and fragments with a length or equivalent spherical diameter of <5 mm, while those <1 μm are classified as nanoplastics. Based on their origin and formation pathways, MPs are categorized into two types: (1) primary MPs, directly released into the environment, and (2) secondary MPs, derived from the fragmentation, abrasion, UV degradation, biodegradation, or photo-oxidation of larger plastic debris. Emerging pollution sources suggest that environmental MP loads will continue to rise. Even if plastic emissions were halted, the secondary degradation of existing plastic waste would persistently augment MP quantities. The United Nations Environment Programme (UNEP) has identified MPs as the “PM2.5 of aquatic environments”.

**Figure 1:**
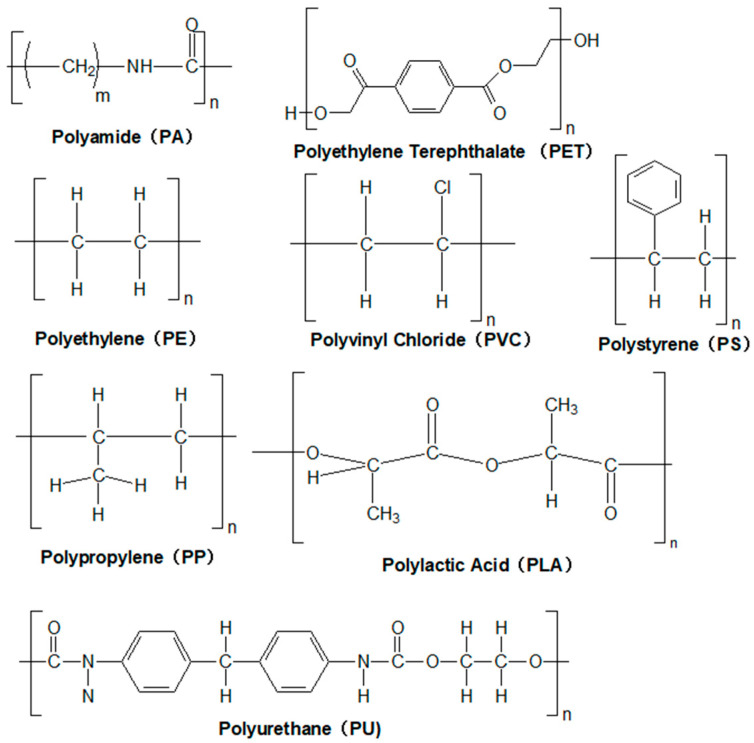
Molecular structures of common MP polymers.

MPs are fundamentally composed of polymeric matrices characterized by diverse and intricate structures. These polymers are formed through the covalent bonding of monomeric units into linear or cross-linked polymeric chains. Variations in polymer types—such as polyethylene (PE), polypropylene (PP), and others—dictate their distinct physicochemical properties, which in turn govern their environmental behavior and fate. Representative structural configurations of common MP polymers are illustrated in Figure 1.

Records of MPs in drinking water are constantly being expanded and updated, which serves as a critical pathway for human exposure to these contaminants. Senathirajah et al. [3] synthesized data from 59 studies to evaluate human’s MP intake, revealing an average annual ingestion of 9029~174,959 MP/L per person via drinking water, which is equivalent to a mass of 25.3–489.7 g, with a mean value of 257.5 g. Bottled water, often perceived as a “safer” alternative, has also been found to contain MPs, influenced by packaging materials, production processes, and storage conditions [3,4,5]. The European Food Safety Authority posits that MPs larger than 150 μm are unlikely to be absorbed by the human body, while those smaller than 1.5 μm can penetrate organs [6]. Recent studies demonstrate that MPs can traverse biological barriers, infiltrating multiple physiological systems, including the digestive system [7], circulatory system [8], excretory system [9], and maternal–fetal interfaces [10]. Notably, MPs have recently been identified in human bone marrow [11]. Although the long-term health implications remain unclear, emerging evidence suggests potential adverse effects on human health.

Drinking water safety is intrinsically linked to public health. The growing public awareness of water quality has intensified the scrutiny of MP contamination in drinking water. Despite extensive research on this topic, the systematic synthesis of findings remains limited, hindering a comprehensive understanding of current research trends and challenges. First, the identification of primary and secondary sources of MPs in drinking water remains fragmented, with limited systematic analysis of contributions from source water pollution, treatment plant inefficiencies, pipe corrosion, and packaging leaching. Second, conventional drinking water treatment processes were not designed for MP removal, and their performance varies widely depending on the particle size, shape, and surface properties, with microfibers and nanoplastics often evading capture. Third, analytical challenges, including low detection limits, matrix interference, and a lack of standardized methodologies, have led to inconsistencies in the MP quantification and characterization across studies, complicating the intercomparison of results.

To address these gaps, this review aims to synthesize the current state of knowledge on MPs in drinking water. Specifically, we (1) systematically identify and evaluate the major sources of MPs in drinking water, from the source water to point-of-use; (2) assess the performance of conventional and advanced water treatment technologies for MP removal, highlighting factors influencing efficiency and limitations; (3) summarize and compare existing analytical methods for MP detection, quantification, and characterization; and (4) critically discuss the potential human health risks associated with MP exposure via drinking water, as well as future research priorities.

## 2. Sources and Fate of Microplastics in Drinking Water Supply Chain

**Figure 2:**
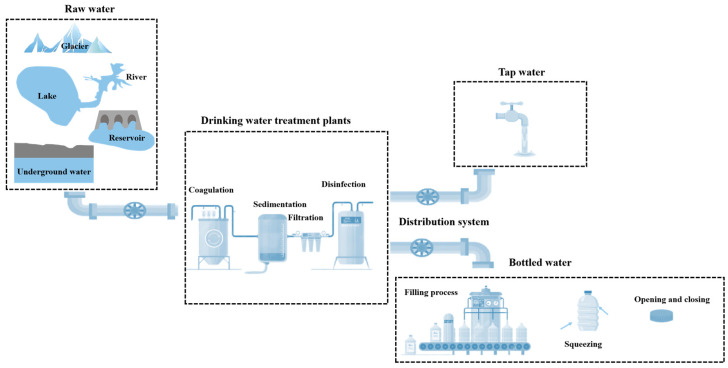
Sources and migration of MPs in drinking water.

Tracking and identifying the sources of MPs in drinking water, as well as mapping their migration across the drinking water supply chain (DWSC), represents a multifaceted challenge. MP contamination exhibits multi-source characteristics, with release occurring throughout the lifecycle of plastic materials, and their presence can be detected across all stages of the DWSC—from raw water sources to end-user consumption (Figure 2). The shapes of MPs primarily include fibrous, fragmental, film-like, foamy, and granular forms. There is significant methodological heterogeneity in the current field of MP research. This heterogeneity is not only reflected in differences in sampling strategies and sample pre-treatment procedures but, more crucially, in the divergent definitions of particle size ranges for MP analysis. Some studies use the abundance of MPs larger than 1 μm as an indicator to characterize drinking water pollution levels [12,13], while others only include particles larger than 20 μm in their abundance statistics [14,15]. Such methodological inconsistency directly hinders the cross-comparison of results from different studies and may introduce false-positive errors due to limitations in detection ranges or variations in operational procedures, thereby impeding the accurate assessment of the actual level of MP pollution in drinking water.

### 2.1. Inputs from Raw Water Sources

Raw water sources (surface water and groundwater) are primary reservoirs of MPs, with contaminants originating from tires, road markings, marine coatings, synthetic textiles, personal care products, plastic pellets, and urban dust. These particles enter aquatic environments via three main pathways: treated wastewater discharge [16], atmospheric deposition [17], and surface runoff [18]. In addition, combined sewer overflow events triggered by heavy rainfall directly discharge insufficiently treated plastic-containing wastewater into aquatic environments, acting as an episodic supplementary pathway for MP input [19].

Groundwater, protected by geological strata, exhibits lower MP contamination compared to surface water [14,20]. Some authors have found MP concentrations from groundwater with values lower than 0.2 MPs/L [21,22]. In addition, open groundwater exhibits a greater diversity of MP colors and larger particle sizes than closed groundwater [23].

Wastewater treatment plants (WWTPs) play dual roles as barriers and emission sources. During treatment, MP-containing wastewater undergoes primary, secondary, and tertiary processes, with approximately 90% transferring to sludge [24]. However, trace amounts of MPs are still discharged into aquatic environments, with Wolff et al. [25] reporting daily emissions of 3000–5900 MPs/L from a German WWTP. Treated wastewater is occasionally used for agricultural irrigation, while sludge is managed through landfilling, composting, or incineration, which leads to MPs from wastewater and sludge migrating into soil, subsequently migrating to surface or groundwater via runoff.

The fate and transport of MPs in raw water are governed by multiple factors, including the particle size, seasonal variations, morphological characteristics, wind velocity, external pressures, and wave energy [26]. Hydrodynamic forces, physical abrasion, thermal fluctuations, and the water column depth accelerate their mobilization into fluvial and lacustrine systems [27]. Buoyant low-density plastics accumulate at water surfaces, enhancing their exposure to photodegradation and chemical weathering, thereby influencing their environmental fate [27]. The socio-economic characteristics, the aquatic environments, physical–chemical characteristics, and hydrodynamic conditions could alter the spatial distribution of MPs in surface water [28]. Li et al. [29] conducted a reanalysis of 53 studies (19 lakes and reservoirs and 35 rivers) and revealed that Asian rivers, lakes, and reservoirs had more MP pollution (2.6 × 10^3^ items·m^−3^) than sources from Europe (1.4 × 10^2^ items·m^−3^) and North America (2.7 × 10^2^ items·m^−3^).

### 2.2. Generation and Transformation During Drinking Water Treatment

Drinking water treatment plants (DWTPs) are designed to purify water but can also be a point of MP generation and transformation. Drinking water treatment processes cannot fully remove MPs, with superior removal efficiencies observed for larger particles (>500 μm). However, the fragmentation of these macroplastics during treatment generates smaller MPs, leading to an increased proportion of smaller particles in effluent compared to influent. Dronjak et al. [15] reported that the proportion of 20–50 μm MPs rose from 8% in influent to 15% in effluent, while larger particles (500–2000 μm) decreased from 19% to <1%. Similarly, Wu et al. [30] demonstrated a significant increase in granular MPs ≤ 20 μm post-treatment.

The infrastructure may also act as an endogenous source of MP pollution. Membrane filtration systems, widely implemented in global water treatment facilities as final barriers against micropollutants, utilize synthetic organic membranes composed of polyethersulfone (PES), PVC, PP, and polyvinylidene fluoride (PVDF). The structural degradation of these membranes may release MPs into treated water [31]. Ding et al. [32] analyzed membrane integrity risks from prolonged operational stresses (physical cleaning, chemical agents, mechanical strain, aging, and abrasion), concluding that aged membranes can release nanoplastics and MPs into distribution networks based on material properties and degradation mechanisms. MPs released from the membrane system originated not only from the membrane material and its additives but also from plastic-made equipment and even the other polymers used in the system [33]. Chu et al. [34] observed that while only nylon and PET were detected in raw water, PVDF emerged post-coagulation/sedimentation, with PVC and PP further identified in treated effluent. The presence of PP and PMMA may originate from polyacrylamide-based flocculants [35].

### 2.3. Release from Distribution Network

As a critical component of the drinking water supply chain, water distribution systems serve as both the terminal barrier for water quality assurance and a critical carrier for the endogenous release of MPs. Pipes and fittings, predominantly made of PVC, PE, PA, and PP, exhibit corrosion resistance but may undergo minor fragmentation and abrasion during water transport, releasing MP particles into drinking water [14,36,37,38]. Beyond mechanical abrasion, interactions between disinfectants and plastic pipes can exacerbate MP release, modulated by the material properties, hydraulic pressure, temperature, pH, disinfectant concentration, and exposure duration [34,39]. The presence of PE pipes with an age of more than 10 years had a considerable effect on the abundance of MPs in the distribution system [6].

MPs released from water distribution systems exhibit significant characteristics of particle size differentiation and regional heterogeneity. An investigation by Tong et al. [40] across 38 tap water samples across Chinese cities revealed maximum MP concentrations of 1247 MPs/L (mean: 440 MPs/L); 1–50 μm particles predominated (78.2%), followed by 100–300 μm (12.1%), 300–500 μm (6.5%), and 500–5000 μm (3.2%). Further analysis demonstrated that a daily consumption of 1500 mL of water would result in an average intake of 660 MP particles per person [40]. Temporal variations in MP concentrations were observed, attributed to variations in the water quality and the heterogeneous distribution of these microparticles in tap water [41].

### 2.4. Release from Bottled/Barreled Water Packaging

Intrinsic release from packaging materials constitutes the primary source. The plastic bottle/barrel bodies are mainly composed of polycarbonate, PE, PET, and HDPE, while the bottle caps are typically made of PS, HDPE, and LDPE [42]. The deformation of plastic water bottles during transportation, usage, and capping due to mechanical effects can also lead to MP release. Studies confirm that the polymer types of bottles and caps correspond to the dominant MP polymers found in bottled water [4,43,44]. Bottle cleaning processes and mechanical actions during filling further contribute to MP contamination. Weisser et al. (2021) [45] collected five sample types from four mineral water bottling lines: raw water, de-ironed water, cleaned post-washing bottles, filled bottles, and capped products. Results showed that MP concentrations increased from <1 MP/L to 317 ± 257 MP/L during bottling, with 81% identified as PE, which is highly consistent with the materials of production equipment. Environmental stress during transportation and storage drives MP release. Vibrations cause friction between bottles/barrels and caps, as well as wear at the interface between barreled water and water dispensers. Temperature fluctuations disrupt polymer molecular forces, triggering MP shedding. Dissolved CO_2_ in carbonated beverages increases internal pressure, amplifying stress on bottle walls and enhancing MP shedding [44], which explains the higher MP levels in carbonated water compared to still water.

Additional factors like sunlight exposure and the chemical properties of the water further contribute to MP contamination [5]. Rigid plastics tend to release larger plastic particles, while deformable plastics and weakly alkaline pH values increase the number of smaller-sized plastics [5]. The frequent opening and closing of bottle caps generates mechanical stress, causing particles or fragments from both the bottle body and caps to detach and enter the water, increasing MP contamination [46,47]. Notably, reusable PET water bottles undergo more cycles of washing, filling, and mechanical stress due to their repeated use, resulting in a typically significantly higher abundance of MP release compared to single-use PET bottles [4,44]. Even in glass-bottled water, MPs primarily originate from abrasion at the interface with plastic caps, causing an increasing number of MPs to enter the water, highlighting the cross-contamination risks associated with mixed-material packaging [4,44]. MPs in glass-bottled water are higher than that in single-use PET bottles [4].

## 3. Microplastic Removal in Drinking Water Treatment

While current water treatment processes have not generally established dedicated removal units targeting MPs, various conventional treatment units can exert a synergistic removal effect on MPs through their inherent functions. Various treatment processes exhibit distinct removal efficiencies depending on their mechanisms and operational conditions (Table 1).

### 3.1. Coagulation/Sedimentation

Coagulation is the first step in the coagulation process, referring to the destabilization of colloidal particles and their initial aggregation into smaller clusters. Flocculation involves the addition of flocculants, which cause the initially aggregated particles to coalesce into larger flocs through bridging, net capture, and electrostatic neutralization, thereby accelerating the sedimentation rate of the particles. Due to the diverse shapes and low density of MPs in water, they cannot be completely removed through the coagulation–sedimentation process. Pivokonsky et al. [12] found that the overall removal efficiency of MPs in water treatment plants was 88%, with the coagulation process accounting for approximately 70.5% of the total removal efficiency, followed by the deep bed filtration and the granular activated carbon filtration, which contributed 22.7% and 6.8%, respectively. Mao et al. [51] used a random forest to quantify the relative importance of factors influencing the MP removal by coagulation: MP shape > coagulant type > coagulant dosage > MP concentration > MP size > MP type > pH.

Aluminum salts and iron salts are commonly used coagulants in WWTPs. Aluminum salts are reported to be more effective than iron salts in removing MPs from water through aggregation and solidification. For example, Wang et al. [52] compared the removal effects of low-molecular-weight metallic salt coagulants and high-molecular-weight polymeric metallic salt coagulants on MPs and found that when the coagulant was 50 mg·L^−1^, the removal efficiency of PS was in the following order: PAC (86.1%) > FeCl_3_ (85.6%) > PFS (65.1%) > alum (50.4%) > Al_2_(SO_4_)_3_ (41.3%) > FeSO_4_ (25.5%). However, under acidic and neutral conditions, Fe13 has a higher net capture and electrostatic neutralization capacity than Al13, achieving an 80% removal rate for PS [53]. Additionally, some researchers believe that the removal mechanisms of inorganic coagulants are similar, and thus the removal effects of aluminum salts and iron salts on PS are not significantly different [54,55]. Fibrous MPs have a larger surface area and therefore a higher removal rate than other shapes of MPs [39]. Moreover, MPs with rough surfaces are more asymmetric and have stronger adsorption forces, making them easier to remove through coagulation [56]. Some researchers argue that larger MPs are more easily removed [54,56,57]. However, other researchers believe that smaller MPs are more easily removed [58,59], as the flocs formed by aluminum salts and iron salts are only a few hundred micrometers in size and cannot capture larger MPs at the millimeter level [29]. The removal of larger MPs is related to the adsorption capacity of flocs for particles, while the removal efficiency of small-sized MPs is predominantly governed by adsorption and electroneutralization interactions with coagulants [60]. Additionally, changes in the physicochemical properties of MPs during aging, such as the color, morphology, size, crystallinity, charge, hydrophobicity, and surface functional groups, also affect the removal efficiency of MPs during coagulation–sedimentation [61]. Aged MPs exhibit superior removal efficiency compared to pristine MPs, as new functional groups (such as -OH, -COOH, and -C=C-) formed on the surface of aged MPs enhance their interactions with coagulants [59].

### 3.2. Filtration

In water treatment processes, granular filter media, such as quartz sand, anthracite, and ceramic particles, are commonly used to retain suspended particles in water. Studies have confirmed that sand filtration has a lower removal rate for smaller MPs but a higher removal rate for larger ones. According to Na et al. [57], the sequential process of coagulation/sedimentation and sand filtration could completely remove 45 μm and 90 μm MPs, whereas 1.2% of 20 μm MPs and 16.6% of the 10 μm MPs passed through the sand media. The removal mechanism of MPs < 10 μm involved interception, capture, entanglement, and adsorption [62]. The presence of biofilms in natural environments aids in the deposition of MPs in porous media, possibly due to the hydrogen bonds that can form between the O-H and N-H groups on cell surfaces and plastic particles [63]. The removal efficiency of MPs of different shapes also varies during sand filtration. For example, Wang et al. [48] studied the characteristics of MPs in each treatment process of an advanced DWTP and found that after sand filtration, the removal efficiencies for fibrous, spherical, and fragment-shaped MPs were 30.9–49.3%, 23.5–50.9%, and 18.9–27.5%, respectively. Other studies have also found that fragment-shaped MPs are more easily removed than fibrous ones during filtration processes [12]. In summary, the removal of MPs during filtration is influenced by multiple factors, and the mechanisms and influencing factors of MP removal during filtration in DWTPs still require further investigation.

The removal of MPs by sand filtration is limited. The addition of biochar to sand filtration systems can enhance the removal performance of MPs. For example, Wang et al. [64] added biochars to a sand column (accounting for 70% of the total column height) and found that 95% of MPs with a particle size of 10 μm could be retained in the column. Biochar derived from lignin and cellulose has a more complex surface morphology and thus exhibits a higher capture capacity for MPs [65].

Membrane filtration is commonly used for the advanced treatment of drinking water and is classified into microfiltration, ultrafiltration, and nanofiltration based on the pore size of the membrane. It is worth noting that the particle size of MPs is larger than that of ultrafiltration membranes, so ultrafiltration can effectively remove MPs from water. However, during the filtration, since the average pore size of the membrane is smaller than that of MPs, a large number of MPs pose the risk of surface contamination and pore clogging, thereby reducing the performance of the membrane filtration [58]. MPs larger than the membrane pore size can be easily repelled by coagulation–ultrafiltration membranes [66]. The separation of microfiltration and ultrafiltration is based on the mechanisms of intermolecular repulsion and electrostatic attraction between MPs and the membrane surface [66]. Once implementing microfiltration and ultrafiltration for MP removal, the strict control of membrane fouling is imperative [67]. The separation mechanism in these processes relies on the interplay between intermolecular repulsion and electrostatic attraction at the membrane–MP interface [66]. Notably, MPs may engage in π-π conjugation with humic substances present in raw water, followed by surface attachment through carboxyl and carbonyl linkages. These interactions facilitate the formation of composite foulants that exacerbate scaling phenomena and induce membrane pore blockage [68]. The study by Li et al. [69] showed that MPs have a synergistic effect with substances in raw water, exacerbating membrane fouling, and that transmembrane pressure is positively correlated with the load of MPs. Therefore, how to effectively alleviate membrane fouling is a key issue that needs to be urgently solved in membrane filtration processes.

### 3.3. Disinfection

Chlorination, ozonation, and ultraviolet radiation are commonly employed disinfection methods in DWTPs. MPs can be degraded or fragmented into smaller sizes and even converted into nanoplastics. The effectiveness of different disinfection methods in degrading MPs varies. Ozonation was more effective in removing nanoplastics, achieving 99.9% degradation and 42.7% mineralization within 240 min, whereas chlorination only reached 7.1% degradation and 4.3% mineralization [70].

Ozone, a strong oxidant, can roughen the surface of MPs and introduce oxygen-containing functional groups, thereby increasing their hydrophilicity and facilitating further oxidative degradation into products such as formic acid and phenol [70]. Ozone oxidation can kill chlorine-resistant microorganisms by attacking their cell membranes [71]. However, MPs can interact with ozone, reducing the number of ozone molecules available to react with bacteria and leaving unaffected pathogens in the water [72]. It has been reported that ozonation can increase the removal rate of MPs to 89.9% [73]. Nevertheless, Pivokonsky et al. [12] reported that ozone treatment in DWTPs reduced the abundance of MPs from 243 ± 17 MPs/L to 224 ± 3 MPs/L, achieving a removal rate of only 7.8%. Additionally, some studies have suggested that ozonation can accelerate the aging and catalytic breakdown of MPs, leading to their fragmentation into smaller particles and fibers under external stress [30,64].

Chlorination is the most widely employed disinfection method. During the chlorination process, MPs undergo substitution or oxidation reactions with hypochlorous acid (HOCl), resulting in the degradation of their original functional groups. The chlorine-induced oxidation of MPs primarily proceeds through two pathways: (1) HOCl decomposition generates molecular oxygen that facilitates MP oxidation, and (2) HOCl generates reactive oxygen species (HO·) and chlorine radicals (Cl·) that interact with MPs, forming carbonyl groups on their surfaces and enabling subsequent auto-oxidation processes [74]. The removal efficacy is influenced by multiple factors, including the chlorine concentration, contact time, water quality parameters, and MP characteristics [75]. Kelkar et al. [76] reported that PP, HDPE, and PS exhibit chlorine resistance with minimal alterations to their chemical and physical structures. However, contradictory findings by Miao et al. [77] demonstrated significant morphological and functional group modifications in MPs during chlorination. Chlorine resistance varies among polymer types, showing an ascending order of PE > PLA > PS when exposed to chlorine concentrations ranging from 2.5 to 5000 mg·L^−1^ [78]. The increase in small-sized MPs may originate from the fragmentation of larger particles during chlorination processes [64].

Photochemical reactions during UV disinfection enhance MPs’ brittleness by reducing elasticity and promoting light absorption [79]. UV disinfection facilitates a 1% removal efficiency through the oxidative breakdown of MPs into smaller fragments [80]. Abrasion, increased hydrophilicity, surface oxidation, the accumulation of oxygen-containing functional groups, and dechlorination can be observed during MP aging. These transformations collectively amplify specific surface areas, shift the organic matter equilibrium toward net release over adsorption, and establish MPs as functional precursors for disinfection byproduct formation [81]. Low-dose UV irradiation increases oxygen-containing functional groups on MP surfaces, whereas a high-dose UV exposure degrades C=O bonds [82].

Current research on MP removal during disinfection predominantly focuses on surface characteristics. However, in DWTPs, co-existing constituents such as heavy metals, organic matter, and particulate matter may interfere with disinfectant–MP interactions. Consequently, the impacts and mechanistic pathways of MPs before and after drinking water disinfection remain insufficiently characterized, necessitating comprehensive investigations into their environmental behavior and transformation dynamics under complex aqueous matrices.

### 3.4. Advanced Microplastic Removal Technologies

Some research efforts have been explored in recent years to develop advanced techniques for the removal of MPs from water, demonstrating considerable potential in both efficacy and innovation. Advanced treatment technologies enhance the overall MP removal, particularly for smaller particles [50,64]. The MP removal rate reached 83% under ozonation combined with granular activated carbon (GAC), which is higher than the removal rate of MPs in conventional water treatment plants (73.3%) [50]. Advanced oxidation processes refer to the generation of highly reactive hydroxyl radicals (•OH) to oxidize and degrade various organic pollutants [83]. Electrochemistry-driven techniques (e.g., electrocoagulation, electroadsorption, electrokinetic separation, and electrochemical degradation) have attracted significant attention due to their high efficiency, operational simplicity, and environmental compatibility [84,85]. Among these, electrocoagulation uses a sacrificial metal anode to electrically generate coagulants in situ and has emerged as a particularly robust and convenient process. Laboratory studies by Perren et al. [86] demonstrated that electrocoagulation can achieve removal efficiencies exceeding 90% for PE, with a peak efficiency of 99.24% observed at pH 7.5.

Magnetic extraction represents another promising approach which uses magnetic seeds and acid with an external magnetic field to improve the separation speed. Nearly a 90% removal of 100–1000 nm particles and a 100% removal of MPs sized 2–5 mm can be achieved by magnetizing MPs with a simple 2-inch permanent NdFeB magnet [87]. Additionally, novel adsorbent materials show great promise. Recently, Kollofrath et al. [88] designed a buoyancy-driven hybrid hydrogel (BDS-gel) that functions as a self-regulating shuttle, capable of transporting and decomposing MPs without external intervention. In simulated seawater, it can degrade 98.7% of polystyrene with only 2 h of light irradiation and maintain efficacy after more than three cycles of reuse.

Although these emerging technologies demonstrate excellent potential for MP removal at laboratory- and even pilot-scale stages, the vast majority still encounter significant challenges in terms of cost-effectiveness, process stability, byproduct control, and scalability. Currently, most have not yet achieved a mature or widespread application in large-scale DWTPs. Future research should focus on advancing the translation of these technologies from laboratory settings to engineering applications, as well as exploring integrated solutions that combine multiple techniques in a synergistic manner to address MP pollution in an economically efficient and effective manner.

## 4. Identification of Microplastics

MPs exhibit high heterogeneity, potentially comprising one or multiple polymer types, resulting in variability in the size, morphology, and chemical composition. Primary detection methodologies include microscopy, spectroscopy, and thermal analysis, each with distinct advantages and limitations.

### 4.1. Microscopy Techniques

Microscopic methods utilize optical or electron microscopy to magnify and analyze MP particles/fibers, determining their shape, size, color, and surface features. Scanning electron microscopy (SEM) and atomic force microscopy (AFM) achieve spatial resolutions of 30 nm and 20 nm, respectively, enabling the precise differentiation of MPs from inorganic particulates. Fluorescence microscopy coupled with dye staining enhances the detection specificity, where stained MPs emit green fluorescence [89]. However, this approach risks false-positives. SEM combined with energy-dispersive X-ray spectroscopy (EDS) provides chemical and morphological characterization, distinguishing carbon-dominant MPs from mineral particles [90]. AFM generates high-resolution 3D topographical maps of MP surfaces with nanoscale precision.

### 4.2. Spectroscopic Techniques

Spectroscopic methods identify the polymer composition by comparing sample spectra against reference libraries. MP characterization predominantly utilizes mid-infrared spectroscopy (4000–400 cm^−1^; 2.5–30 μm wavelength range) [91]. Fourier-transform infrared spectroscopy (FTIR) exploits vibrational/rotational absorption bands of chemical bonds to generate polymer-specific “fingerprints,” enabling the identification of particles >10 μm. A quantitative analysis is feasible via absorption peak intensity/area measurements. Mukotaka et al. [41] applied FTIR to trace MP sources in treated water, detecting post-treatment contaminants such as >100 μm polyester fibers and >50 μm PVC fragments. Raman spectroscopy offers superior sensitivity, detecting particles down to 1 μm (or ~300 nm with confocal microscopy) [21]. Surface-enhanced Raman spectroscopy (SERS) employs noble metal nanostructures to amplify Raman signals by 106–1014-fold via localized surface plasmon resonance, enabling nanoplastic detection [92].

### 4.3. Thermal Techniques

A thermal analysis identifies polymers via degradation byproducts by measuring physicochemical property changes under controlled heating, though its destructive nature imposes limitations on the sample recovery and subsequent analysis. Principal techniques include pyrolysis–gas chromatography/mass spectrometry (Py-GC/MS) and thermal desorption–gas chromatography/mass spectrometry (ATD-GC-MS). Unlike spectroscopic methods, Py-GC/MS requires a minimal sample pre-treatment and effectively analyzes <1 μm. However, its destructive protocol and small sample capacity (~0.5 mg) restrict high-throughput applications. ATD-GC-MS combines a thermogravimetric analysis (TGA) with thermal desorption. Degradation products and oligomers are captured on sorbent traps, desorbed at elevated temperatures, and analyzed via GC-MS. Compared to Py-GC/MS, ATD-GC-MS accommodates complex matrices and larger sample masses (up to 100 mg), enabling nanoplastic polymer identification. However, it demands higher MP concentrations (>1% *w*/*w*) and a ~200× greater sample mass.

Studies on the occurrence characteristics of MPs in drinking water reveal significant methodological discrepancies in detection outcomes. For example, Kirstein et al. [6] assessed the MP contamination in drinking water using u-FTIR and Py-GC/MS. Both methods successfully determined the content of MPs in drinking water, with a range of 0–0.022 ± 0.019 MPs/L. However, the polymer types identified by the two methods were not consistent. u-FTIR identified eight polymer types, including PA, PES, acrylic compounds, PVC, PS, and, the relatively less common, PE, PU, and PP. In contrast, Py-GC/MS only detected five polymer types: PA, PES, PVC, PS, and PP. It is evident that a single detection method often has limitations and cannot comprehensively and accurately reflect the full picture of MPs. Therefore, it is recommended to integrate various detection methods to obtain valuable complementary information and effectively compensate for the shortcomings of a single technology. Moreover, the limitations of existing detection technologies are particularly evident when analyzing MPs in complex environmental samples. On the one hand, the sample pre-treatment process is complex, usually requiring multi-stage filtration and manual sorting, which is not only time-consuming and labor-intensive but also prone to human error. On the other hand, the spectral characteristics of MPs are susceptible to interference from organic matter coverage or pigments, and the interpretation of characteristic peaks highly depends on the experience of the operator, which greatly restricts the accuracy and reliability of the detection results. In view of this, machine learning, as a technology with powerful data processing and analysis capabilities, provides a new solution to the above problems. Machine learning can be combined with detection technologies to significantly improve the accuracy and detection efficiency of MP analysis.

## 5. Potential Risk of Microplastics

**Figure 3:**
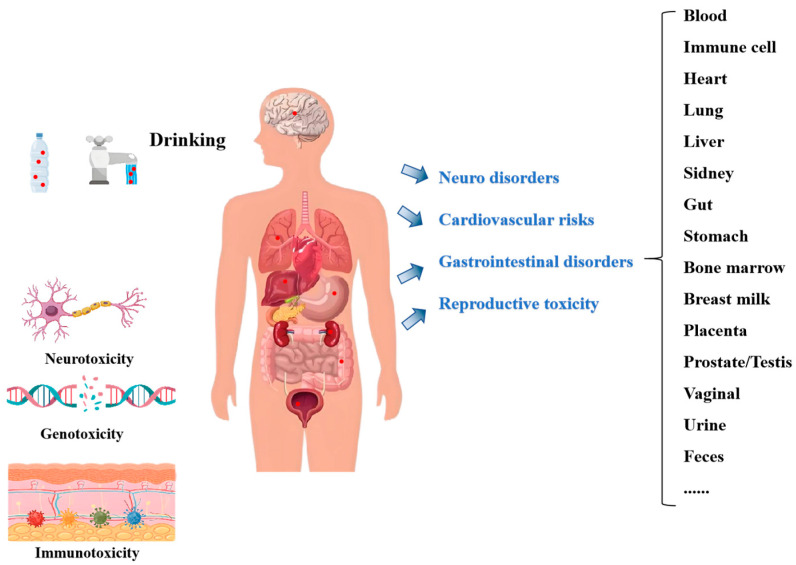
Impact of MPs on health.

The physicochemical properties of MPs play a significant role in influencing human health. For instance, smaller-sized particles and ultrafine fibers are considered relatively more hazardous types of MPs to humans. The health hazards posed by MPs can be categorized into direct and indirect hazards (Figure 3). Direct hazards refer to the immediate physical damage caused by the MPs themselves, such as physiological injuries to various organs due to the ingestion of MP particles. Indirect hazards pertain to the risks associated with additives used in the plastic manufacturing process, as well as the potential for MPs to act as carriers that adsorb toxic chemicals, thereby posing risks to human health. In regions where drinking water has a low abundance of MPs, some scholars argue that MPs do not pose significant health problems to humans [93,94]. However, the potential health risks associated with MPs should not be overlooked [93,94].

### 5.1. Direct Hazards

MPs with smaller particle sizes are more readily absorbed by the human body. It has been reported that MPs smaller than 150 μm can easily traverse the gastrointestinal epithelium, MPs of approximately 10 μm in size can cross the placental and blood–brain barriers, and even smaller MPs of 2.5 μm can reach the systemic circulation through endocytosis [95]. Huang et al. [96] demonstrated that, under normal breathing rates, MPs can cover half of the nasal cavity’s inner surface. Larger-sized MPs are more likely to deposit rapidly in the upper respiratory tract, while smaller nanoplastics are more capable of escaping or reaching deeper respiratory levels. Once absorbed by the human body, MPs can cause various types of physiological damage, such as disrupting immune function, causing obstruction and vascular inflammation through internalization, pulmonary arterial hypertension, systemic inflammatory responses, and blood cell toxicity. They can also directly or indirectly alter the human metabolism and energy balance and induce oxidative stress effects leading to cytotoxicity, neurotoxicity, reproductive toxicity, and carcinogenicity [97].

Wu et al. [98] investigated the cytotoxicity and efflux pump inhibitory capacity of two different sizes of PS particles on human colon cancer Caco-2 cells. They found that 5 μm PS particles induced stronger effects than 0.1 μm PS particles. Moreover, high concentrations of 5 μm PS particles could reduce the activity of ABC transporters by inducing mitochondrial depolarization and potential ATP depletion. Jeon et al. [99] discovered that polystyrene exhibited a more significant toxicity to THP-1 macrophages compared to PP. PS nanoparticles (50 nm) could cross the blood–brain barrier and accumulate in the brain, activating microglial cells and causing severe neuronal damage [100]. Lin et al. [101] observed that 80 nm PS particles entering human normal liver cells and human normal lung epithelial cells could induce mitochondrial dysfunction, such as mitochondrial damage, excessive mitochondrial reactive oxygen species, changes in the mitochondrial membrane potential, and the inhibition of mitochondrial respiration. A recent study indicated that the concentration of MPs/nanoplastics in the brain is significantly higher than that in the liver and kidneys [102].

### 5.2. Indirect Hazards

The precursor plastics of MPs contain a vast array of chemical substances, among which a common category is additives, including plasticizers, flame retardants, colorants, stabilizers, and so on. Compared with other additives, plasticizers (especially phthalates and bisphenol A) have been more extensively studied for their hazards. Eker et al. [103] revealed a positive correlation between serum bisphenol A levels and non-functional adrenal incidentalomas. Polybrominated diphenyl ethers (PBDEs), which serve as flame retardants, can trigger oxidative stress, disrupt hormones, and induce molecular carcinogenesis in tissues. Moreover, exposure to both MPs and flame retardants may enhance oxidative stress-mediated neurotoxicity in mice [104].

MPs can adsorb other pollutants from the environment, such as heavy metals and organic pollutants, and may subsequently interact with them in a synergistic or antagonistic manner. This can alter the overall toxicity and physicochemical properties, thereby increasing the risk of human disease. Particularly, smaller-sized MPs, with their larger surface area-to-volume ratio, possess a stronger capacity for adsorbing and releasing pollutants, as well as penetrating and disrupting cells and tissues. These chemicals can interfere with the human endocrine system, causing hormonal imbalances that may affect the immune system, reproductive system, and other physiological functions of the human body. Deng et al. [104] demonstrated that MPs contaminated with phthalates led to higher alterations in sperm. PP loaded with 17β-estradiol posed a relatively higher risk of pollutant release in the gastric fluid of marine organisms under constant temperature conditions, potentially causing more severe harm [105]. Moreover, MPs of different particle sizes exhibit varying adsorption capacities. For instance, 70 nm PS has an adsorption capacity for PCBs that is 1–2 orders of magnitude higher than that of 10–180 μm PE [106]. MPs can also act as carriers for a variety of microorganisms. The formation of biofilms on MPs may influence microbial dissemination through microbe–MP–toxic chemical interactions. In drinking water, once microorganisms grow on pipes, biofilms can form on MPs. These biofilms may flake off due to aging and subsequently enter the drinking water.

## 6. Recommendations for Future Research

Despite significant advancements in MP research in aquatic environments, critical challenges persist in comprehensively understanding the distribution, abundance, and composition of MPs in drinking water. To address current methodological limitations, future research should prioritize the following four strategic directions:

(1) Establishing standardized detection procedures and methods for MPs in drinking water. Current studies exhibit pronounced methodological heterogeneity, manifesting in inconsistent sampling strategies, pre-treatment approaches, and detection techniques. These inconsistencies compromise data comparability and introduce false-positive risks. Consequently, it is imperative to establish unified standardized protocols that consider all the steps associated with the quantification and characterization of MPs, ensuring data accuracy, reproducibility, and comparability.
(2) Enhancing treatment processes to remove MPs. Traditional processes in current DWTPs are still insufficient in removing MPs and pose the risk of secondary release. Given the potential threats of MPs to the environment and human health, there is an urgent need to develop more efficient new water treatment technologies to enhance the removal capacity of MPs. Exploring and applying new adsorbent materials that can efficiently capture MP particles in water bodies, as well as the great potential of membrane technology in MP removal, are important directions. Moreover, integrating various water treatment processes to form a comprehensive treatment system is also an effective way to improve the removal efficiency of MPs.
(3) Developing health risk assessment methods for MPs in drinking water. Data on the impact of MPs on human health are relatively limited, but their potential effects should not be overlooked. Drinking water is an important source of human exposure to MPs. By combining the absorption and metabolic conditions of MPs in the human body with toxicological data on MPs, it is necessary to quantify the migration and metabolic patterns of MPs in the human body, analyze their synergistic toxic effects with coexisting pollutants, establish dose–effect relationship models, and comprehensively assess the potential threats of MPs to human health.
(4) Constructing a multi-scale coordinated life cycle prevention and control system for MPs in drinking water systems. Establish multi-level prevention and control barriers from the four dimensions of “source reduction–process interception–end-of-pipe treatment–recycling and regeneration” to form a systematic management plan for the entire chain of “sources–water treatment plants–pipelines-users”, reduce the risk of MPs in drinking water, and ensure public drinking water safety.

## 7. Conclusions

This study presents the current research on MPs in drinking water supply chains, encompassing their sources, fates, removal technologies, detection methods, and potential hazards to human health. MPs in drinking water systems exhibit multi-source input characteristics, originating from environmental infiltration into water sources; leaching from materials in water distribution systems; migration from bottled water packaging interfaces; and re-release during water treatment processes. These pathways collectively contribute to contamination across the entire supply chain, from raw water to end-user consumption. It is noteworthy that current detection technologies are limited by non-standardized sampling procedures and the limitations of identification methods, leading to the questionable comparability of data across different studies. There is an urgent need to establish standardized detection protocols that include quality control systems and to construct a multidimensional detection system through the integration of various technologies. The potential hazards of MPs remain a critical concern. Future work needs to integrate research from environmental science, toxicology, and public health to clarify the dose–effect relationships of MPs, improve risk assessment systems, and promote technological innovation and policy regulation to effectively ensure drinking water safety and public health.

## Figures and Tables

**Table 1:** Summary of MP removal efficiency in DWTPs.

Sources	Detection	Polymer Type	Treatment	Removal Efficiency (%)	References
Czech	SEM + FTIR + Raman spectroscopy	PET, PP, and PE	Coagulation–sand filtration	70.0	[35]
Czech	SEM + FTIR + Raman spectroscopy	PET, PP, and PE	Coagulation–sedimentation–sand filtration–activated carbon filtration	81.0	[35]
Czech	SEM + FTIR + Raman spectroscopy	PET, PP, and PE	Coagulation–dissolved air flotation–sand filtration–activated carbon filtration	83.0	[35]
China	SEM + Raman spectroscopy	PET, PE, PP, PAM, PS, and PVC	Coagulation–sedimentation–GAC filtration–sand filters–ozone tank	88.6	[48]
Switzerland	Nanoparticle tracking analysis (NTA) + turbidimeter	PS	Coagulation–sedimentation–sand filtration–GAC filter	99.2	[49]
China	Micro-Raman spectrometer	PP, PET, PE	Coagulation–sedimentation–sand filtration–ozonation integrated with GAC filtration–disinfection	82.3	[50]
China	Micro-Raman spectrometer	PP, PET, PE	Coagulation–sedimentation–sand filtration–disinfection	73.3	[50]
Iran	SEM + µ-Raman spectroscopy	PP, PET, PE, PS, PTFE, PU	Screen–coagulation–sand filtration–disinfection	50.1	[13]
Iran	SEM + µ-Raman spectroscopy	PP, PET, PE, PS, PTFE, PU	Screen–coagulation–sand filtration–disinfection	48.4	[13]
Iran	SEM + µ-Raman spectroscopy	PP, PET, PE, PS, PTFE, PU	Coagulation–sand filtration–disinfection	55.2	[13]

## Data Availability

All data are included in this article.
